# The burden of moderate-to-heavy soil-transmitted helminth infections among rural malaysian aborigines: an urgent need for an integrated control programme

**DOI:** 10.1186/1756-3305-4-242

**Published:** 2011-12-30

**Authors:** Abdulhamid Ahmed, Hesham M Al-Mekhlafi, Seow Huey Choy, Init Ithoi, Abdulelah H Al-Adhroey, Awatif M Abdulsalam, Johari Surin

**Affiliations:** 1 Department of Parasitology, Faculty of Medicine, University of Malaya, 50603, Kuala Lumpur, Malaysia; 2 Department of Parasitology, Faculty of Medicine and Health Sciences, Sana'a University, Sana'a, Yemen

**Keywords:** Soil-transmitted helminths, risk factors, aboriginal schoolchildren, Malaysia

## Abstract

**Background:**

Soil-transmitted helminth (STH) infections, among the most common neglected tropical diseases, continue to be a major threat to the health and socioeconomic wellbeing of infected people especially children in developing countries.

**Methods:**

A cross-sectional study among 254 aboriginal schoolchildren was conducted in order to determine the current prevalence and intensity of infections and to investigate the potential risk factors associated with moderate-to-heavy burden of STH infections among these children.

**Results:**

Overall, 93.7% of children were found to be infected with one or more STH species. The prevalence of trichuriasis, ascariasis and hookworm infections were 84.6%, 47.6% and 3.9%, respectively. Almost half of the participants had heavy trichuriasis, one-quarter had heavy ascariasis whereas all hookworm infections were light infections. Overall, moderate-to-heavy STH infections accounted for 56.7% of the total infections. Univariate analysis revealed that those using untreated water supply (*P *= 0.013), living in houses without toilets (*P *= 0.027) and having domestic animals in the houses (*P *= 0.044) had significantly higher prevalence of moderate-to-heavy infections than others. Logistic regression analysis confirmed using untreated water for drinking (*P *= 0.001) and the absence of a toilet in the house (*P *= 0.003) as significant risk factors of moderate-to-heavy STH infections among these children.

**Conclusion:**

The high proportion of moderate-to-heavy STH infections further confirms the need for serious attention towards these devastating diseases that has put lives and the future of aboriginal children in jeopardy. Introduction of more poverty alleviation schemes, proper sanitation, provision of clean and safe drinking water, health education, as well as the introduction of periodic school-based deworming programmes are imperative among these communities in order to curtail the transmission and morbidity caused by STH.

## Background

The morbidity caused by soil-transmitted helminths (STH), notably *Ascaris lumbricoides, Trichuris trichiura *and hookworm, has been a matter of great public health concern throughout the world for decades. Recent estimates suggest that nearly two billion people are infected by these worms worldwide [1,2]. Pre-school and school-age children in the impoverished communities with low socioeconomic status are more prone to be infected. Therefore, they tend to suffer most from the adverse health consequences of these infections, which include growth retardation, iron deficiency anaemia (IDA), Vitamin A deficiency (VAD), poor cognitive function, school absenteeism and a dismal academic performance [3-7]. However, these consequences are proportional to the worm burden (intensity of infections); infections of moderate-to-heavy intensity are associated with greater morbidity whereas infections of light intensity are often asymptomatic [7,8]. As the eradication of these infections is not a viable proposition, the WHO aims to curtail the transmission dynamics and to reduce the morbidity caused by the worm burdens on infected individuals [9].

In Malaysia, although great socioeconomic transformation has led to a significant reduction in the prevalence of parasitic infections in urban areas [10,11]. STH infections remain highly common among the poor rural and aboriginal populations [12-17]. The continuance of STHs in these communities over the past decades could be due to the enviroment becoming heavily contaminated with the infective stages of these helminths through indiscriminate open defaecation and poor sanitation and therefore, acquiring the infection can hardly be avoided [18]. In such a situation, a better understanding of the risk factors of moderate-to-heavy burden of STH infections would be pertinent to save the vulnerable population from the adverse effects of this infection. Within this context, this study aimed to determine the current prevalence and intensity of STH infections and to investigate the potential risk factors associated with the moderate-to-heavy burdens of infection among the aboriginal schoolchildren in Satak, Pahang, Malaysia.

## Methods

### Study area and subjects

This cross-sectional study was conducted between July and August 2010 in Satak, Raub district, Pahang, located about 200 kilometers northeast of Kuala Lumpur, the national capital of Malaysia. The area consists of aboriginal settlements, located approximately between longitude 101°37'48"E - 101° 43'47"E and latitude 3°59'22"N - 4°02'03"N. There are five main communities namely; Pos Satak, Ruai Hulu, Ruai Hilir, Sungai Kelang and Sungai Rensong. Houses are made of wood or bamboo in which environmental sanitation, as well as personal hygiene, was poor. Like other parts of Malaysia, this area has an equatorial rain-forest climate with hot, humid conditions and rainfall throughout the year. The vegetation is of the thick rain forest type and there are few water streams in the area.

Sekolah Kebangsaan Satak (the National School of Satak), a primary school for aboriginal children with a total enrolment of 364 pupils, was selected for this study. However, a total of 289 pupils aged 6-13 years participated voluntarily in the study and were interviewed to fill in the questionnaires. The next day, only 254 of them delivered their stool samples for examination. Therefore, statistical analysis for associations between variables was based on this sample size.

### Questionnaire

The children and their parents were interviewed, in their home settings, to fill in a pre-tested questionnaire for their family background and socioeconomic information. The questions were designed to elicit information on the age, sex, family size, household monthly income, parents' educational and employment status, source of drinking water, presence of toilet facilities, as well as data on medical history and personal hygiene practices.

### Parasitology

The children were given labeled, clean, wide-mouthed and screw-capped containers and were instructed to bring their early morning stool samples the next day. The collected stool samples were transported for examination at the stool processing laboratory in the Department of Parasitology, University of Malaya. The samples were examined by using the Kato-Katz technique as described by Martin and Beaver [19] for the presence of soil-transmitted helminths (*A. lumbricoides*, *T. trichiura *and hookworm) eggs. For quality control purposes, duplicate Kato Katz thick smears were prepared for each stool sample. The smears were read twice by two different microscopists. First for hookworm within 30 minutes and second for *Ascaris*/*Trichuris *after 40 minutes or more. An average of the counts were used in this report. To determine the worm burden, egg counts were taken and recorded as eggs per gram of faeces (epg) for each positive sample. Harada-Mori faecal cultivation technique was also used to detect hookworm larva [20]. The results of the Harada Mori technique were consistent with the Kato Katz readings on hookworms. Intensity of infections was graded as heavy, moderate or light according to the criteria proposed by the WHO [21]. Scores for the intensity of infections were given to each STH species (light = 1, moderate = 2 and heavy = 3) and infections with species score ≥ 2 were included in the analysis.

### Statistical analysis

Data analysis was performed using SPSS for Windows software (SPSS version 13). Univariate analysis was used to investigate the association between moderate-to-heavy STH infections as the dependent variable and age, sex, parents' educational and employment status, household monthly income, family size, source of drinking water, presence of toilet facilities, have domestic animals at households, playing with the soil and eating soil (geophagy) as the explanatory variables. For the purpose of data analysis, all the factors in the survey were coded in a binary manner. For example, gender (female = 0, male = 1); age (≥ 10 years = 0, Age < 10 years = 1); STH infections (negative-to-light STH infections = 0, moderate-to-heavy STH infections = 1); presence of toilet in the house (no = 1, yes = 0); educational status of parents (no formal education = 1, ≥ 6 years formal education = 0); household monthly income (< RM500 = 1, ≥ RM500 = 0); source of drinking water (untreated = 1, treated/piped = 0); family size (> 7 members/large = 1, ≤ 7 members = 0); have domestic animals at household (yes = 1, no = 0) and play barefooted with soil (yes = 1, no = 0). *P *< 0.05 was considered statistically significant. Logistic regression was performed to identify the potential risk factors of moderate-to-heavy STH infections; variables that showed an association with *P *≤ 0.20 were used to develop the multivariate regression model as suggested by Bendel and Afifi [22].

### Ethical consideration

The protocol of this study (Reference Number: 788.74) has been approved by the Medical Ethics Committee of the University of Malaya Medical Centre, Kuala Lumpur, Malaysia. During the visits to the school and the villages, community meetings were held with the headmaster of the school, teachers, heads of the villages, the parents and their school-age children before the commencement of the study in order to give a clear explanation of the objectives of the study. The parents and their children were informed that their participation is voluntary and they can withdraw from the study at any time without assigning any reason whatsoever. All the parents had agreed to have their children participate in the study. Verbal consents were obtained.

## Results

### General characteristics of the Subjects

A total of 254 aboriginal schoolchildren (51.2% females and 48.8% males) with a mean age of 9.5 years (SD = 0.20) participated in this study and delivered stool samples for examination. Almost half of the parents had at least 6 years of formal education. Poverty is predominant in the aboriginal communities and about two-thirds of their children belonged to low household monthly income families (< RM500; US$1.00 = RM3.00). The majority of the subjects live in houses without toilets and/or treated water supply. The general demographic and socioeconomic characteristics of the study subjects are shown in Table 1.

**Table 1:** General characteristics of the 254 aboriginal schoolchildren participating in the study

Characteristics	Frequency (%)
**Age groups:**	
≥ 10 years	48.0
< 10 years	52.0
**Gender:**	
Males	48.8
Females	51.2
**Socioeconomic status:**	
Fathers' education level (at least 6 years)	51.2
Mothers' education level (at least 6 years)	46.5
Low household income (< RM500)	63.0
Working fathers	89.6
Working mothers	5.2
Large family size (> 7 members)	22.4
Piped water supply	19.3
Presence of toilet (pit or flush) in the house	48.4

### Prevalence and distribution of STH infections

The overall prevalence of STH infections was 93.7%. The prevalence of trichuriasis, ascariasis and hookworm infections were 84.6%, 47.6% and 3.9%, respectively. Moreover, more than one third of the infected children had mixed infection.

The prevalence of STHs among children aged < 10 years was almost similar to the prevalence among those aged ≥ 10 years (91.7% compared to 95.9%, respectively). Similar results were reported among the males and females (94.4% compared to 93.1%, respectively).

**Figure 1:**
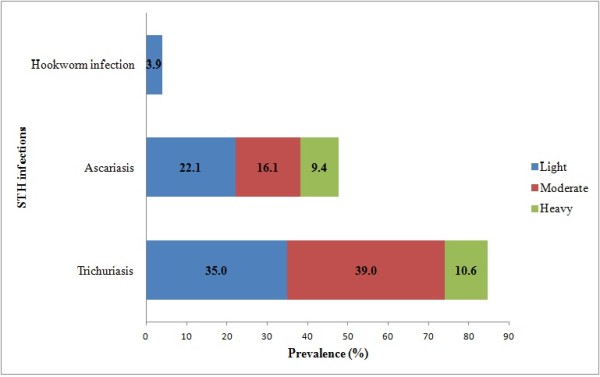
**Prevalence and intensity of infections of ascariasis, trichuriasis and hookworm infections among aboriginal schoolchildren in Satak, Pahang (n = 254)**.

Moreover, 58.6% and 53.3% of the infected children (comprised of 49.6% and 25.5% of the examined subjects) had moderate-to-heavy burden of trichuriasis and ascariasis, respectively, whereas, all cases of hookworm infection were of light worm burden. However, the combined moderate-to-heavy STH infections (trichuriasis + ascariasis) accounted for 60.5% of all the infections (Figure 1).

### Risk factors of moderate-to-heavy STH infections

Univariate analysis results for the association of moderate-to-heavy STH infections with the explanatory variables are illustrated in Table 2. The results showed that the use of untreated water supply (OR = 1.90; 95%CI = 1.14, 4.71; P = 0.013), the absence of toilets (OR = 1.76; 95%CI = 1.07, 2.90; P = 0.027) and having of domestic animals in the household (OR = 3.69; 95%CI = 1.01, 8.24; P = 0.044) showed significant associations with the moderate-to-heavy STH infections.

**Table 2:** Associations of potential risk factors with moderate-to-heavy STH infections among aboriginal schoolchildren in Satak, Pahang (n = 254)

Variables	Moderate-to-heavy STH infections		
	
	Prevalence (%)	OR (95% CI)	*P*
**Age:**			
≥ 10 years	62.3	1.23 (0.98, 1.56)	0.083
< 10 years	51.5	1	
**Gender:**			
Male	59.7	1.27 (0.78, 2.09)	0.348
Female	53.8	1	
**Fathers' education:**			
No formal education	59.7	1.27 (0.77, 2.09)	0.348
≥ 6 years formal education	53.8	1	
**Mothers' education:**			
No formal education	59.6	1.27 (0.78, 2.12)	0.322
≥ 6 years formal education	53.4	1	
**Household income:**			
< RM500/month (low)	59.4	1.34 (0.80, 2.24)	0.260
≥ RM500/month	52.1	1	
**Family size:**			
> 7 members (large)	59.6	1.17 (0.64, 2.13)	0.609
≤ 7 members	55.8	1	
**Toilet in house:**			
No	63.4	1.76 (1.07, 2.90)	0.027*
Yes	49.6	1	
**Source of drinking water:**			
Untreated (river, rain, well)	60.5	1.90 (1.14, 4.71)	0.013*
Treated (Piped)	40.8	1	
**Have domestic animals at household:**			
Yes	58.0	3.69 (1.01, 8.24)	0.044*
No	27.3	1	
**Play barefooted with the soil**			
Yes	60.8	1.55 (0.93, 2.58)	0.093
No	50.0	1	

OR: odds ratio; CI: confidence interval

^* ^Significant association (*P *≤ 0.05)

Logistic regression analysis confirmed that consuming untreated water supply as a source of drinking water and for other daily needs (OR = 2.70; 95%CI = 1.31, 5.81; P = 0.001) and the absence of a toilet in the house (OR = 2.40; 95%CI = 1.30, 4.82; P = 0.003) were the significant risk factors of moderate-to-heavy burden of STH infections among these children (Table 3).

**Table 3:** Results of multivariate analysis of potential risk factors for moderate-to-heavy STH infections among aboriginal schoolchildren in Satak, Pahang (n = 254).

Variables	Moderate-to-heavy STH infections	
	OR (95% CI)	*P*
Source of drinking water (untreated)	2.70 (1.31, 5.81)	0.001*
Toilet in house (no)	2.40 (1.30, 4.82)	0.003*
Have domestic animals at household (yes)	2.53 (0.68, 8.63)	0.152
Age (≥ 10 years)	1.16 (0.57, 1.79)	0.584
Play barefooted with the soil (yes)	1.46 (0.74, 2.84)	0.273

OR: odds ratio; CI: confidence interval

^* ^Significant risk factor (*P *≤ 0.05)

Variables included in the model were age, toilet in house, source of drinking water, have animals at household and playing barefooted with the soil

## Discussion

The present study revealed an alarmingly high prevalence (93.7%) of STH infections among aboriginal children in rural Malaysia and this has caused more concern as the prevalence is consistent with previous studies conducted among aboriginal children in different states of Malaysia over the past decades [12-17]. Despite the significant reduction in the overall prevalence of intestinal parasitic infections in the urban areas [10,11], the trend in rural areas, especially among the underprivileged aboriginal populations, remains largely unchanged with regard to the triad of *A. lumbricoides*, *T. trichiura *and hookworm that continues to afflict these populations at a rate much higher than the threshold recommended by the WHO (20%) for the implementation of mass deworming of schoolchildren in endemic areas [23]. A national worm control programme was launched in 1974 and rural primary schoolchildren, including aboriginal schoolchildren, received a single dose of pyrantel pamoate once or twice a year (24). However, the programme could not continue because of the fact that pyrantel pamoate was not effective against *T. trichiura *and hookworm, hence, the problem persists.

Evidence from previous reports around the world indicated that ascariasis is more prevalent compared to the other helminth infections [1,2]. However, our study proved trichuriasis as the exceptionally most predominant infection in Malaysia which is consistent with many previous reports [14,16,24]. The higher prevalence of *T. trichiura *infection in our survey may represent insusceptibility to the anthelmintic drugs. The adult worm normally lie embedded in the walls of the lumen. Low cure rate of anthelmintic drugs against *T. trichiura *has been reported in Malaysia and abroad (25, 26, 27). Although there is no convincing proof yet on drug resistance, there is a need for a more detailed investigation of whether drug resistance is emerging in *T. trichiura *(27). This alarming fact should be given a major consideration in any de-worming programmes in rural Malaysia. As such it may be mandatory to consider a 3-day course of 400 mg Albendazole which has proven to be very effective against heavy trichuriasis among the aboriginal population in Malaysia [18,28]. The low prevalence of hookworm infection reported in this study is also consistent with previous similar studies in Malaysia [14,17,18]. This could be due to the non-loamy nature of the soil in most parts of the country, which is not favourable for the development of hookworm larvae.

The results from the present study showed that about two thirds of the subjects infected with *T. trichiura *and/or *A. lumbricoides *had moderate-to-heavy worm burdens, and therefore, they are at risk of suffering the consequences of the infections. This figure is no different from the findings reported previously [14,16,18]. Considering the strongly-positive relationship between poverty and the pervasive continuance of STH infections, poverty in these communities may play an important role in the persistence of this problem. Heavily infected individuals usually become lethargic and weak and thus unlikely to gain from benefitting fully from their domestic and school environments. As infection and its negative impacts are likely to continue to adulthood, their social functioning and wage-earning capacity or productivity in later life may be compromised and thereby the population at risk being trapped in a vicious cycle of poverty and disease which may ultimately affect national development [29]. During our constant visits to the study area and interactions with the children, we observed that many of them always looked tired and weak. School absenteeism rate among them was also high. These could be possibly related to the burden of STH infections among them.

Investigating the possible risk factors associated with moderate-to-heavy STH infections among the participants revealed that using untreated water supply for household needs is a significant risk factor of infection. A similar finding was reported in Ethiopia [30]. Untreated water is always likely to be contaminated with parasites eggs and/or cysts in areas with poor sanitation. Hence, its usage for household activities enhances the likelihood for parasitoses. Moreover, the lack of toilet facilities in the house is another significant risk factor identified by the present study; and this is in agreement with previous reports [17,18].

Contamination of the household surroundings with infected faeces due to the lack of toilets increases the chances of infection and re-infection by STH. The eggs/larvae of STH can remain viable in the soil for a very long period of time and could easily get picked-up by man or animals. Hence, proper sanitation (which helps break the helminth transmission cycle) is critical to being healthy and considered as a key component in any intestinal parasite control programme [21]. Moreover, our study demonstrated a significant association between moderate-to-heavy STH infections and the presence of domestic animals in the households. Animals such as cats and dogs move about in the contaminated environment and also mix freely with the members of their households. Others such as chickens mingle with and feed on the excreta deposited by children around the compound and also move freely within the compound. All these could facilitate the spread of the infections.

Great success has been achieved in many countries in reducing the transmission, prevalence and intensity of STH infections by mass chemotherapy hand-in-hand with improvements in social infrastructures, sanitation and poverty alleviation. For instance, in the Republic of Korea, such programmes were able to reduce the prevalence of STH infections from 84.3% in 1971 to about 0.02% in 2004 [31]. A similar story of success was reported in Zanzibar and China [32,33]. Malaysia is witnessing great transformations in terms of socioeconomic and infrastructural developments. Through well planned national control programmes, the country has witnessed great achievements in controlling filariasis and malaria [21]. Similar success can also be achieved by introducing an integrated national helminth control programme, focusing on the vulnerable populations as recommended by the WHO. About 2.5 million rural children attending schools in the country are expected to benefit immensely from this programme when introduced.

## Conclusion

This study reaffirms that STH infections are still prevalent and is a matter of a serious concern in the rural aboriginal communities of Malaysia. There is an urgent need to identify integrated and effective control measures to reduce STH infections significantly in these communities. These measures should focus on reducing poverty, providing proper sanitation, as well as the provision of clean and safe drinking water and proper public enlightenment (health education) regarding good personal hygiene and good sanitary practices. Introduction of school-based de-worming programmes will also help to significantly reduce the prevalence and intensity of the infection in the area.

## Authors' contributions

AA was involved in all phases of the study, including study design, data collection, data analysis, interpretation, and write-up of the manuscript; JS, HMA and II designed and supervised the study, and revised the manuscript. HMA was involved in the statistical analysis of data. SHC, AHA and AMA were involved in the collection and laboratory examination of samples. All authors read and approved the final manuscript.

## Competing interests

The authors declare that they have no competing interests.

## Acknowledgements

The authors wish to acknowledge the support and cooperation of the Headmaster, staff and pupils of the National School of Satak in making this survey successful. We also wish to express our appreciation to the parents, and the staff of the District Education Office in Raub, for their kind support and cooperation. This study was supported by the University of Malaya research grants (PS181/2009C & PS233/2010B).
